# Hysterical Men

**Published:** 1889-05-25

**Authors:** 


### Within the Wards.

### HYSTERICAL MEN.

Hysteria is popularly associated with voluntary weakness of mind. Many medical men of the less thoughtful sort also incline to this idea, and if they had their way would prescribe the dashing of a bucket of cold water over every patient suffering from a hysterical attack. But there are hysterics and hysterics just as there are old soldiers and old soldiers. An attack of hysteria may be as genuine and as uncontrollable as a headache or a sleepless night. The human nervous system is a most peculiar, most delicate, and most variously sensitive organism. The sanest man in the world might, under appropriate conditions, become a lunatic. Similarly, though women to a large extent monopolise hysteria, we may have genuinely hysterical men. According to a paper read by Mr. Adami at the Cambridge Medical Society, hysteroid fits are not at all rare in lads and young men between the ages of sixteen and twenty-five, the most common period being from eighteen to twenty-two. The symptoms are not unsimilar to those in females, though, as might be guessed, less dramatic and pronounced. On the contrary, the male hysteric is often phlegmatic and unemotional, inclined rather to stupidity and silence. Of the two, this is perhaps preferable. Various paralytic seizures of a strictly temporary character may affect a hysterical man. He may lose the use of an arm, or of a hand or finger, or of one or both legs, or he may be quite unable to speak above a whisper, or even to speak at all. The limbs, instead of being paralysed, may be contracted and contorted in a variety of ways. Sensibility to pain may be lost in some parts of the body, whilst in others it may be very excessive. The cure of some of these attacks, especially those affecting the mind of the male hysteric, is very simple, and is not unlike in rationale to the "bucket of cold water" method favoured by old-fashioned practitioners. It consists in taking the terminal phalanx of the patient's little finger between the thumb and first finger of the operator and pressing the nail downwards and backwards. The effect is promptly and decidedly painful, and the attack of hysteria is usually as promptly cured—often to the loudly expressed disgust of the patient. Of course, all cases cannot be treated in this rough-and-ready way; and no class of patients is better fitted to test the discernment and skill of the physician than the infinite variety of those, both male and female, who may occasionally suffer from hysteria.

